# Pancreatic cancer stem cells may define tumor stroma characteristics and recurrence patterns in pancreatic ductal adenocarcinoma

**DOI:** 10.1186/s12885-021-08123-w

**Published:** 2021-04-09

**Authors:** Gokce Askan, Ibrahim Halil. Sahin, Joanne F. Chou, Aslihan Yavas, Marinela Capanu, Christine A. Iacobuzio-Donahue, Olca Basturk, Eileen M. O’Reilly

**Affiliations:** 1 Memorial Sloan Kettering Cancer Center, 300 East 66th street, office 1021, New York, NY 10065 USA; 2 H. Lee Moffitt Cancer Center and Research Institute, Tampa, USA; 3 David M. Rubenstein Center for Pancreatic Cancer, New York, USA; 4 Weill Cornell Medical College, New York, USA

**Keywords:** Pancreatic ductal adenocarcinoma, Pancreatic cancer, Cancer stem cells, Tumor stroma, Sonic hedgehog, Desmoplasia, Tumor microenvironment, CD44, ESA, Recurrence pattern

## Abstract

**Background:**

Herein, we investigate the relationship between pancreatic stem cell markers (PCSC markers), CD44, and epithelial-specific antigen (ESA), tumor stroma, and the impact on recurrence outcomes in pancreatic ductal adenocarcinoma (PDAC) patients.

**Methods:**

PDAC patients who underwent surgical resection between 01/2012–06/2014 were identified. CD44 and ESA expression was assessed by immunohistochemistry. Stroma was classified as loose, moderate, and dense based on fibroblast content. Overall survival (OS) and relapse-free survival (RFS) were estimated using the Kaplan-Meier method and compared between subgroups by log-rank test. The association between PCSC markers and stroma type was assessed by Fisher’s exact test.

**Results:**

*N* = 93 PDAC patients were identified. The number of PDAC patients with dense, moderate density, and loose stroma was 11 (12%), 51 (54%), and 31 (33%) respectively. PDAC with CD44^+^/ESA^−^ had highest rate of loose stroma (63%) followed by PDAC CD44^+^/ESA^+^ (50%), PDAC CD44^−/^ESA^+^ (35%), CD44^−/^ESA^−^ (9%) (*p* = 0.0033). Conversely, lack of CD44 and ESA expression was associated with the highest rate of moderate and dense stroma (91% *p* = 0.0033). No local recurrence was observed in patients with dense stroma and 9 had distant recurrence. The highest rate of cumulative local recurrence was observed in patients with loose stroma. No statistically significant difference in RFS and OS was observed among subgroups (*P* = 0.089).

**Conclusions:**

These data indicate PCSCs may have an important role in stroma differentiation in PDAC. Our results further suggest that tumor stroma may influence the recurrence pattern in PDAC patients.

**Supplementary Information:**

The online version contains supplementary material available at 10.1186/s12885-021-08123-w.

## Background and materials

Pancreatic ductal adenocarcinoma (PDAC) is a highly aggressive cancer and there are significant unmet therapeutic needs in the management of this malignancy. PDAC accounts for 3% of all new cancers and is the 4th leading cause of cancer-related death in men and women in the United States (US) [1]. A future projection suggests that if the mortality rate of PDAC continues in the current direction, it will be the second leading cause of cancer-related death in the US by 2030 [2]. At the current time, key therapies for metastatic PDAC are cytotoxic-based approaches, including FOLFIRINOX (5-fluorouracil, leucovorin, irinotecan, and oxaliplatin) [3] and gemcitabine and nab-paclitaxel [4], and both regimens improve survival compared to single-agent gemcitabine; however median survivals remain less than a year [3].

With progress in the understanding of the molecular underpinnings of cancer biology and development, targeted therapies and immunotherapy have led to dramatic improvements in survival outcomes of selected solid tumors [5]. However, unfortunately, these approaches including immunotherapy have achieved limited success in the management of PDAC due in part to its distinct molecular behavior [6, 7]. For example, immune checkpoint inhibitors have been shown to be effective in patients with mismatch repair deficient (MMR-D) PDAC [8], which accounts for about 1 % of PDAC cases [9, 10]. Recently, a phase III trial of olaparib, a poly (ADP-ribose) polymerase (PARP) inhibitor, reported improvement in progression-free survival (PFS) in *BRCA*-mutant metastatic PDAC patients when administered as maintenance therapy following platinum-based treatment, compared to placebo [11]. Notably, *BRCA* gene mutations are also relatively uncommon and seen in about ~ 5–7% of unselected PDAC patients [12–14] making PARP inhibitors, a promising therapeutic strategy, applicable to a minority of patients with metastatic PDAC.

The PDAC microenvironment, which is a focus of interest for the development of therapeutic agents, carries very sophisticated biologic features [15]. Stromal desmoplasia is a complex connective tissue reaction leading to dense stroma and hypoxic tumor microenvironment. Clinical and preclinical studies have suggested that desmoplasia in the PDAC microenvironment is driven mainly by the sonic hedgehog pathway (SHH) [16] which led to a series of clinical trials targeting this pathway. Unfortunately, studies of SHH inhibitors have not shown benefit and notably, one of the SHH inhibitors led to detrimental outcomes in PDAC patients [17]. These disappointing findings have resulted in significant loss of interest in SHH inhibitors and most recently, other stroma modifying agents including PEGPH20, a pegylated version of hyaluronidase, was investigated in metastatic PDAC patients. The combination of this agent with FOLFIRINOX and gemcitabine nab-paclitaxel chemotherapies did not result in any survival benefit and led to detrimental outcomes in patients who received PEGPH20 with concurrent FOLFIRINOX, [18–20].

Pancreatic cancer stem cells (PCSC) are enriched with SHH signaling which is one of the signaling pathways that induces of desmoplastic reaction in PDAC. Notably, a preclinical study identified a 46-fold increase in SHH signaling in PCSCs which were observed to express CD44, epithelial specific antigen (ESA), and CD24 as compared to normal pancreatic epithelial cells [21]. However, the loss of ESA has been associated with the epithelial-mesenchymal transition which is associated with cancer stem cell generation [22], the dual interaction between tumor stroma and PCSC has not been well-investigated and it is unclear if PCSC may have a role in defining the composition of tumor stroma and disease behavior. In this study, we investigated the relationship between the PCSC markers and tumor stroma and evaluated the site of first recurrence pattern by stroma characteristics in PDAC patients who underwent surgical resection. We also examined the potential impact of stroma type and PCSC on survival outcomes in our cohort.

## Method

**Fig. 1:**
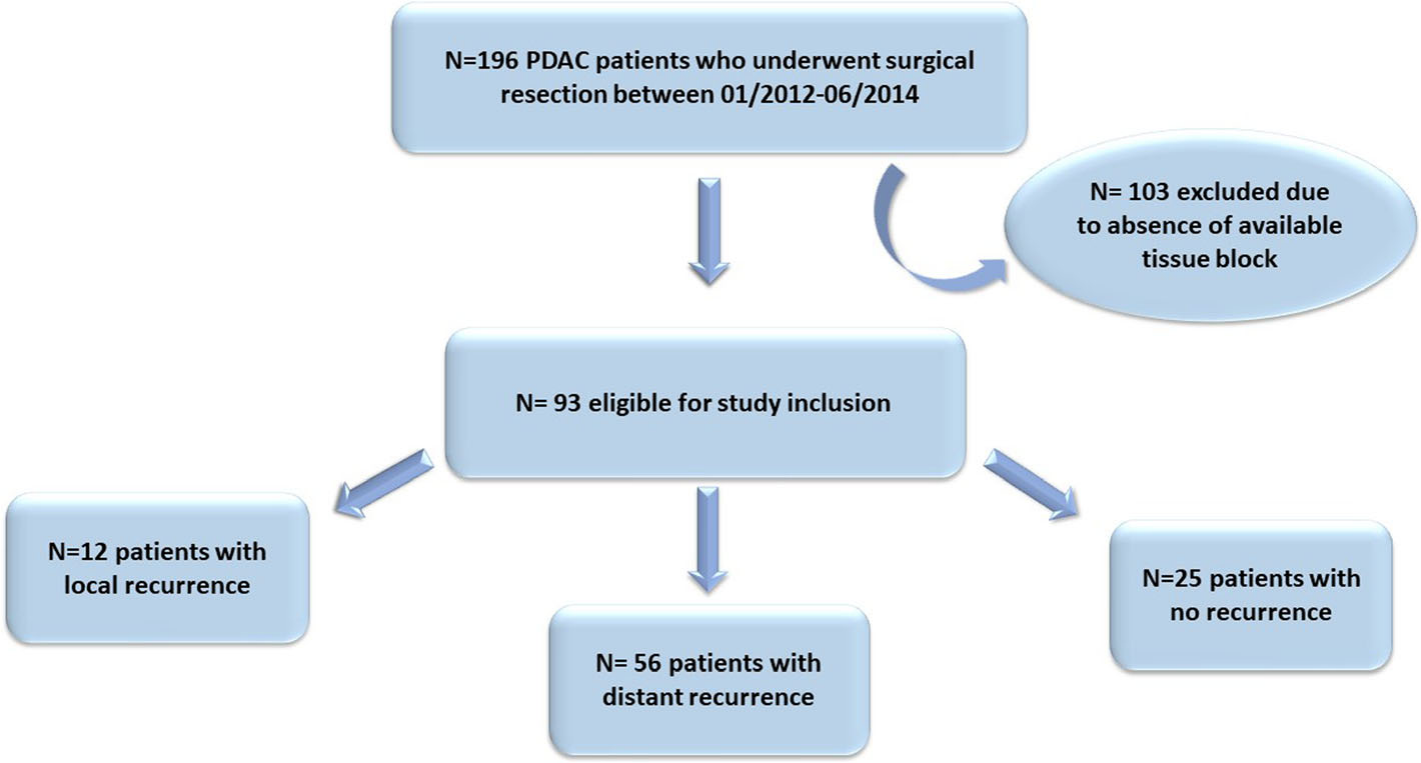
Patient Disposition

With the approval of the Institutional Review Board, PDAC patients who underwent surgical resection at Memorial Sloan Kettering (MSK) between 01/2012–06/2014 were identified by the query of the institutional cancer database (Fig. 1). All available tissue blocks with PDAC diagnosis regardless of recurrence status were retrieved from the Department of Pathology. All slides in each case were re-reviewed by a pathologist (GA) for confirmation of the diagnosis and for selection of the best representative tumor block on which to perform immunohistochemistry. Patients whose tumor had predominant fibrosis with low tumor cellularity due to neoadjuvant therapy were excluded from the study. The American Joint Committee on Cancer pancreatic cancer staging 8th edition was used for TNM staging of patients. Available medical records were reviewed to obtain clinical, imaging, and pathological data, including information on age, gender, stage of disease, grade of tumor, vascular and perineural invasion, type of surgery, radiological findings for site of first recurrence pattern, and follow-up and survival status. The first recurrence site (metastatic vs local) was used to define the recurrence pattern in our study.

### Statistical analysis

The characteristics of our cohort are reported using frequency and percentages for categorical variables including clinical and pathological covariates. Mean and standard deviation were used in continuous variables. A composite stem cell marker (C+/E+, C+/E-, C−/E+, and C−/E-) was derived using CD44 and ESA biomarkers. The association between stem cell markers and stroma type was assessed by Fisher’s exact test.

Overall survival (OS) and relapse-free survival (RFS) were defined as time from diagnosis to death and time from diagnosis to the first recurrence respectively. Patients alive at the time of analysis in 02/2019 were censored. OS and RFS were estimated using the Kaplan-Meier method and compared by the log-rank test. A Cox proportional hazards model was used to evaluate the association between stroma types and stem cell markers on outcomes. The model was further adjusted with nodal status as it is one of the known confounders in this disease.

Cumulative incidences of any, local and distant recurrences were estimated using competing risks methods and compared between stroma types using Gray’s test. All statistical analyses were performed using SAS Version 9.3 (SAS Institute, INC., Cary, NC, USA) or R version 3.5.1 (R Foundation for Statistical Computing, Vienna, Austria) using the ‘cmprsk’ package. All *p*-values were two-sided. *P*-values of < 0.05 were considered to indicate statistical significance.

### Immunohistochemistry

The best representative formalin-fixed paraffin-embedded tissue section was chosen for each case. Three micrometer thickness sections were obtained and underwent overnight deparaffinization at 37 °C. The sections were submerged for antigen retrieval in citrate buffer (ph 6.0). Immunohistochemical staining was performed with the streptavidine biotin peroxidase method by using antibodies as follows: CD44 (156-3c11,1:3000, Cell Signaling), ESA (EpCAM) (BerEP4, 1:4, Ventana). Positive control tissues for CD44 and ESA were HeLa cells and normal colonic tissue respectively.

**Fig. 2:**
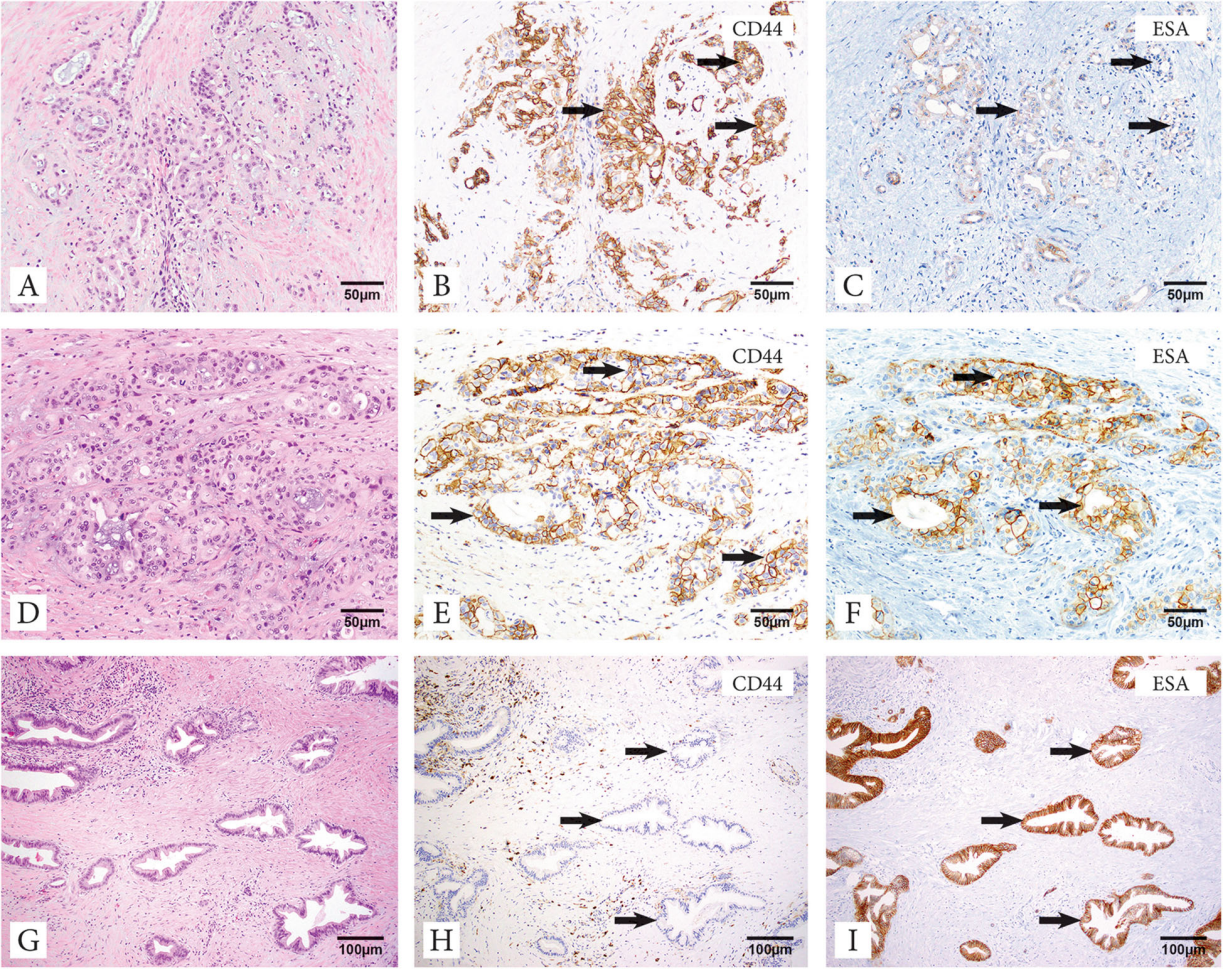
The relationship between tumor stroma and cancer stem cell markers. PDAC with loose stroma with H&E staining (**a**) positive for CD44 staining (**b**), negative for ESA staining (**c**). PDAC with moderate stroma with H&E staining (**d**) positive both for CD44 staining (**e**) and ESA staining (**f**). PDAC with dense stroma with H&E staining (**g**) negative for CD44 staining (**h**), positive for ESA staining (**i**). The arrows indicate the tumor cells which show positive or negative staining for CD44 and ESA

Only membranous staining was regarded as expression (Fig. 2). Antibody expression was categorized into 5 groups according to the percentage of positive tumor cells: 0, none; 1, 1–10%; 2, 11–50%; 3, 51–80%; 4, 81–100%. Staining intensity was scored as 0, none; 1, weak; 2, moderate; 3, strong (Figure 1S). The total scores (0–12) were averaged and the score was considered positive when average score > median. Stroma was classified as loose, moderate density, and dense based on fibroblast content using Hematoxylin and Eosin (H&E) stain [23, 24] (Fig. 2).

## Results

A total of 196 PDAC patients who underwent surgical resection of their primary tumor at MSK were identified between 01/2012–06/2014. Among these, 103 patients were excluded due to lack of available tissue block (Fig. 1). Ninety-three patients were included in the final analysis. The clinicopathological features are summarized in Table 1. Most patients were male (65%) and presented with a pancreatic head tumor (82%). Notably, 97% of the cohort had T3 disease and 71/93 (76%) had lymph node metastasis with N1 or N2 disease. Five patients in our cohort received neoadjuvant chemotherapy. More than half of the tumors (53/93) were either moderate to poorly differentiated or poorly differentiated. Of the 93 tumors analyzed for stroma type, 31 (33%) had loose stroma, 51 (55%) had moderate stroma and 11 (12%) had dense stroma. The frequency of tumors with CD44^+^/ESA^+^, CD44^+^/ESA^−^. CD44^−^/ESA^+^, CD44^−^/ESA^−^ were 16/93 (17%), 19/93 (20%), 36/93 (39%), and 22/93 (24%) respectively (Table 1).

**Table 1:** Clinicopathological Featurse of Cohort (*N* = 93)

Gender	N (%)
Female	33 (35)
Male	60 (65)
**Tumor Grade**	
Well or moderatetly differentiated	40 (43)
Moderate to poor	25 (27)
Poor	28 (30)
Stage at Diagnosis*	
pT1	1 (1)
pT2	2 (2)
pT3	90 (97)
**Tumor Location**	
Head	76 (82)
Body	8 (8)
Tail	9 (10)
**Lymphovascular Invasion**	
Present	78 (84)
Absent	15 (16)
**Perineural Invasion**	
Present	88 (95)
Absent	5 (5)
Lymph Node Status*	
No lymph nodes (N0)	22 (24)
N1	35 (38)
N2	36 (38)
**Margin status**	
Positive	23 (25)
Negative	70 (75)
**History of neoadjuvant therapy**	
Yes	6 (6)
No	87 (94)
**History of adjuvant therapy**	
Yes	77 (88)
No	11 (12)
**Cancer Stem Cell Markers**	
CD44^+^/ESA^+^	16 (17)
CD44^+^/ESA^−^.	19 (20)
CD44^−^/ESA^+^	36 (39)
CD44^−^/ESA^−^	22 (24)

*The American Joint Committee on Cancer pancreatic cancer staging 8th edition was used for TNM staging. T1: tumor ≤ 2 cm, T2: tumor 2–4 cm, T3:tumor > 4 cm, T4 > tumor extends into nearby major blood vessels; N1:1–3 regional lymph nodes, N2:> 4 regional lymph nodes

The relationship between tumor stroma type and clinical characteristics is summarized in Table 2. The T stage of disease, which may impact the recurrence pattern particularly local recurrence [25], was similar across subgroups and the frequency of T3 disease in loose, moderate, and dense stroma tumors was 97, 96, and 100% respectively. Lymph node metastasis (N1 + N2) was observed in 65, 82, 81% of the patients with loose, moderate, and dense stroma respectively. PDAC patients with a loose stroma pattern had more moderate to poor or high-grade tumors (71%; 22/31) as compared to PDAC patients with moderate (53%; 27/51) or dense stroma (36%; 4/11). The tumor stroma was also examined by the expression status of cancer stem cell markers, CD44 and ESA. The relationship between tumor stroma and expression of cancer stem cell markers is shown in Fig. 2 and summarized in Table 3. We observed significantly different stroma type among subgroups determined by the expression of cancer stem cell markers particularly by CD44 status (*p* = 0.0033). For example, the percentage of loose stroma was highest in CD44^+^/ESA^−^ tumors (63%), followed by CD44^+^/ESA^+^ (50%) tumors and while loose stroma was relatively uncommon in CD44^−^ tumors; 25% in CD44^−^/ESA^+^ and 9% in CD44^−^/ESA^−^ tumors. Conversely, the highest rate of moderately dense or dense stroma was observed in CD44^−^ tumors; CD44^−^/ESA^−^ (91%; 20/22) CD44^−^/ESA^+^ tumors (75%; 27/36). CD44^−^ tumors also had the highest rate of well to moderately differentiated tumors as compared to CD44^+^ tumors (Table 1S).

**Table 2:** Relationship between Tumor Stroma and Clinical Features

	Loose stroma N (%)	Moderate stroma N (%)	Dense stroma N (%)
**History of neoadjuvant therapy**			
Yes	2 (6)	4 (8)	–
No	29 (94)	47 (92)	11 (100)
**History of adjuvant chemotherapy**			
Yes	24 (89)	42 (84)	11 (100)
No	3 (11)	8 (16)	–
**History of adjuvant radiotherapy**			
Yes	3 (11)	12 (24)	4 (36)
No	25 (89)	38 (76)	7 (64)
**Lymphovascular invasion**			
Present	23 (74)	44 (86)	11 (100)
Absent	8 (26)	7 (14)	–
**Perineural invasion**			
Present	28 (90)	50 (98)	10 (91)
Absent	3 (10)	1 (2)	1 (9)
**Margin status**			
Positive	8 (26)	12 (24)	3 (27)
Negative	23 (74)	39 (76)	8 (73)
**Stage**			
pT1	1 (3)	–	–
pT2	0	2 (4)	–
pT3	30 (97)	49 (96)	11 (100)
**Node Status**			
N0	11 (35)	9 (18)	2 (18)
N1	11 (35)	19 (37)	5 (45)
N2	9 (30)	23 (45)	4 (36)
**Grade**			
Well or moderately differentiated	9 (29)	24 (47)	7 (64)
Moderate to poor, poorly differentiated	22 (71)	27 (53)	4 (36)
**Cumulative dstant metastasis by site**	14 (45)	33 (65)	9 (82)
**- Lung**	–	5 (15)	4 (44)
**- Liver**	9 (64)	18 (55)	4 (44)
**- Lung + liver**	4 (29)	4 (12)	–
**- Other**	1 (7)	6 (18)	1 (12)

**Table 3:** Relationship Between Tumor Stroma and CD44 and ESA Expression

Stroma	CD44+/ESA- N (%)	CD44−/ESA+ N (%)	CD44+/ESA+ N (%)	CD44−/ESA- N (%)	
**Loose**	12 (63)	9 (25)	8 (50)	2 (9)	**P = 0.0033**
**Moderate**	5 (26)	21 (58)	7 (44)	18 (82)	
**Dense**	2 (11)	6 (17)	1 (6)	2 (9)	

**Fig. 3:**
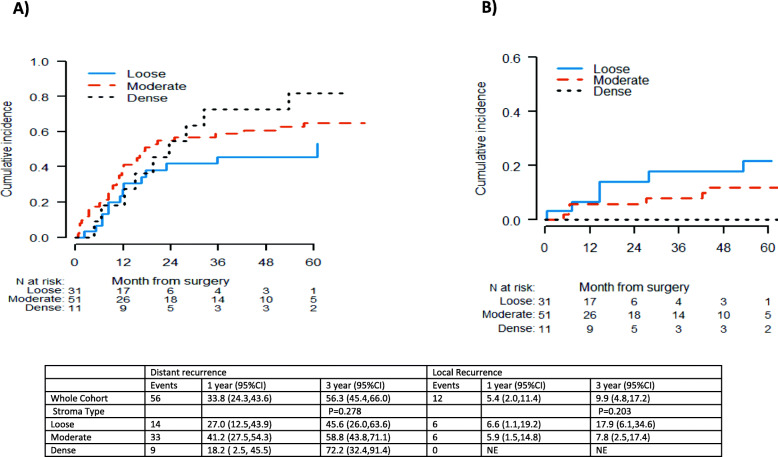
Time to Recurrence by stroma type: **a** Distant recurrence; **b** Local recurrence

**Fig. 4:**
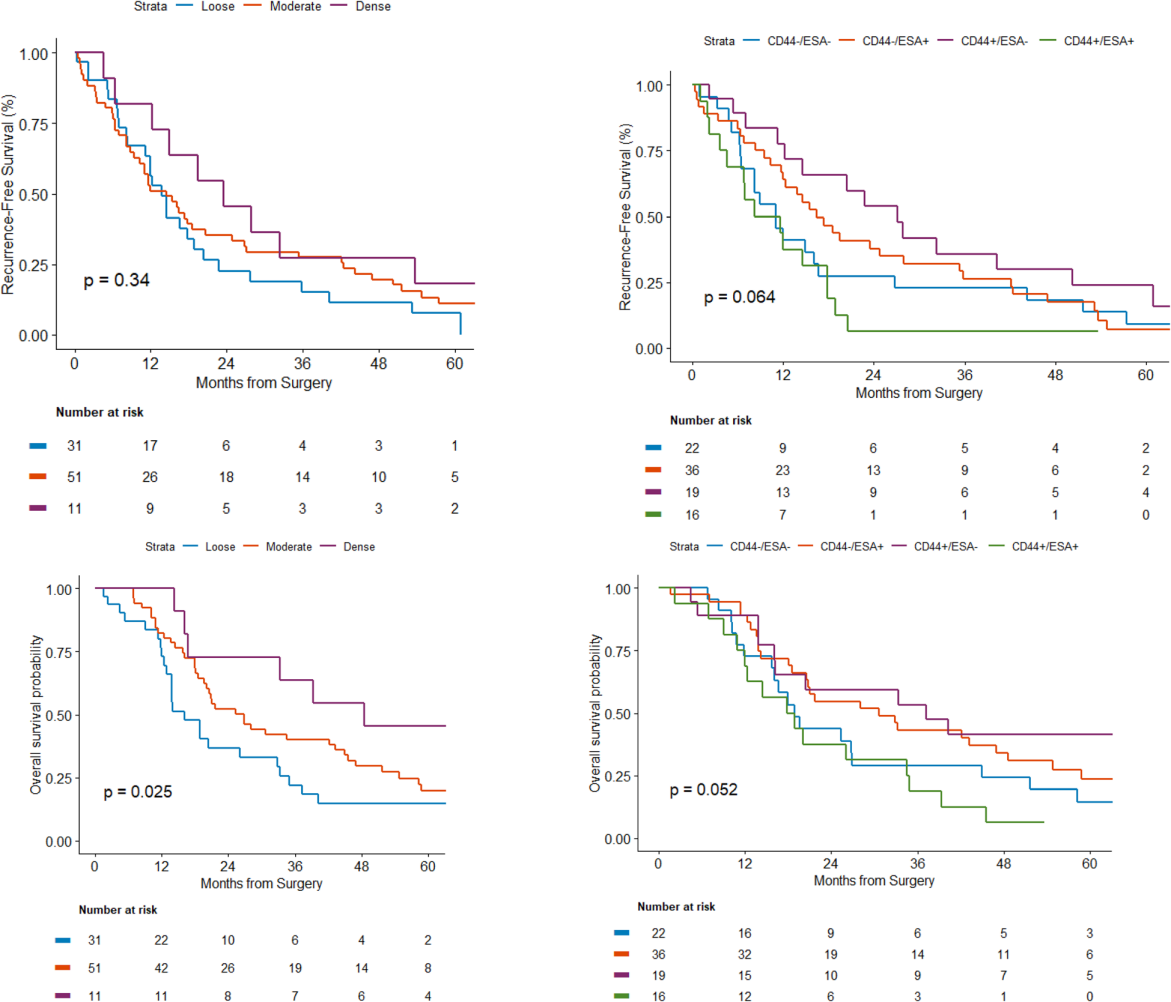
Survival Outcomes by stroma type and stem cell markers

The median follow-up among surviving patients from surgery was 20 months. At the time of analysis, we observed 68 recurrences. Cumulative incidence at 1-year and 3-year post-surgery were 39% [95%CI: 30–49%] and 66.3% [95%CI: 55–75%], respectively. Local recurrence was observed in 12 patients and none was observed among patients with dense stroma and highest occurrence in loose stroma, however, the difference in the cumulative incidence of local recurrence did not translate into statistical significance (*p* = 0.203) (Fig. 3). Cumulative incidences of local recurrence at 1 and 3 years were 7% [95%CI: 1–19%] and 18% [95%CI: 6–35%], respectively for loose stroma subtype (*n* = 31). Cumulative incidence of distant recurrence at 1-year and 3-year post-surgery was 34% [95%CI: 24–44%] and 56% [95%CI: 45–66%], respectively. We did not observe a significant difference between stroma types and time to distant recurrence (*p* = 0.278) although, the highest rate of distant recurrence at 3-years was observed in patients with dense stroma (72.2, 95%CI:32.4–91%) (Fig. 3, Table 4). Patients with loose stroma had a significantly shorter median OS as compared to patients with dense stroma with a median OS estimate of 16.1 [95%CI: 12.4–32.8] vs 48.5 [95%CI: 16.0-NR] months (*p* = 0.025) (Fig. 4). However, this significance became borderline after adjustment for nodal status of disease (HR = 1.66; 95% CI 0.97–2.82; *p* = 0.061). No statistically significant association between PSCS markers and OS and RFS outcomes was identified (Table 5).

**Table 4:** Association between Tumor Stromal Types and Recurrence Pattern

Stroma type	n (%)	Local Recurrence n (%)	1 Year (95% Cl)	3 Year (95% Cl)	***p*** value	Distant Recurrence n (%)	1 Year (95% Cl)	3 Year (95% Cl)	***p*** value
**Loose**	31 (33)	6 (19)	6.6 (1.1, 19.2)	17.9 (6.1, 34.6)	0.203	14 (45)	27.0 (12.5, 43.9)	45.6 (26.0, 63.3)	0.278
**Moderate**	51 (55)	6 (12)	5.9 (1.5, 14.8)	7.8 (2.5, 17.4)		33 (65)	41.2 (27.5, 54.3)	58.8 (43.8, 71.1)	
**Dense**	11 (12)	–	–	–		9 (82)	18.2 (2.5, 45.5)	72.7 (32.4, 91.4)

**Table 5:** Median Overall Survival by Stroma and Cancer Stem Cell Markers

	Estimate	Lower Limit	Upper Limit	***P*** value
**Stroma**				
Loose	16.1184	12.3684	32.8289	***P*** **= 0.0255;** *After adjusted by N status ***P*** **= 0.0613, HR: 1.67 (95%C: 0.0.98–2.92)**
Dense	48.5526	16.0197	.	
Moderate	26.8092	18.5855	43.2237	
**Stem Cell Markers**				
C+/E+	18.3717	10.8553	34.4737	**P: 0.0517** *After adjusted by N status; P = 0.089; HR:0.50 (95%CI: 0.23–1.1)
C+/E-	37.2039	13.8816	.	
C−/E+	30.6579	18.5855	46.9079	
C−/E-	18.8816	11.8421	26.9079	

C*: CD44, E*: ESA (Epithelial specific antigen), N: Lymph node status

We did not observe a significant association between stroma and RFS. Median RFS for patients with loose, moderate density and dense stroma, was 13.8 [95%CI: 8.3–18.8], 23.5 [95%CI: 6.4–53.6], 14.5 [95%CI: 8.8–20.6] months respectively (*p* = 0.33). The median RFS was numerically shorter in patients CD44+/ESA+ and CD44−/ESA- tumors and following adjustment for N status of disease no statistically significant association was observed (Data not shown).

## Discussion

Stem cells represent approximately 0.5–1% of pancreatic cancer cells and with their self-renewal capacity, they have an important role in the development and progression of PDAC [21]. However, their role in tumor stroma differentiation and clinical outcomes is unclear. In our study, we identified a significant difference in the distribution of stroma types among PDAC patients by PCSC markers. The expression of CD44, a mesenchymal adhesion molecule [26], was associated with loose stroma while CD44 negative tumors had more frequent moderate density and dense stroma, indicating the gaining of stemness features may be linked to loose tumor stroma, which is associated with adverse survival outcomes in our study,. Although it did not reach statistical significance, we observed differences in recurrence patterns among stroma type in PDAC patients who underwent surgical resection of their primary tumor. We identified a higher incidence of distant recurrence in patients with dense stroma compared to patients with loose stroma, however that did not translate into statistical significance perhaps due to the small size of the cohort. We observed no local recurrence among patients with dense stroma while more local events occurred in patients with loose stroma and again this did not reach statistical significance. Notably, after adjusting for nodal status, there was a trend to worse survival outcomes in patients with loose stroma while cancer stem markers were not associated with either RFS or OS (Table 5); these findings are limited due to the size of the overall cohort.

Our findings suggest that there may be an interaction between PDAC tumor stroma, and PCSC. Firstly, dense stroma with desmoplastic features in PDAC patients does not appear to be associated with worse outcomes in our study population. In fact, we observed a trend to improved survival outcomes in PDAC patients with dense stroma as compared to PDAC patients with a loose stroma pattern. Although our findings are limited by the small size of our cohort, they are consistent with recently growing evidence supporting the observation that dense stroma may restrain the tumor epithelial component and could be a reactive response to suppress local tumor invasion [27, 28]. Consistent with this observation, although it did not reach statistical significance, we further observed that patients with loose stroma had a higher cumulative incidence of local recurrence within 3 years post-surgery as compared to patients with a moderate stroma. At the time of analysis, none of the patients with dense stroma developed local recurrence; all had distant recurrence. Our data suggests that a desmoplastic reaction may function as a physical barrier. Importantly, in our cohort, patients with loose stroma had higher-grade tumors as compared to patients whose tumors had a dense stroma pattern suggesting more aggressive tumors may develop in the relatively less fibrotic environment. Our findings are also consistent with the lack of improvement in survival outcomes in PDAC patients treated with stroma targeting agents, particularly with SHH inhibitors [29]. Notably, at the time of analysis, among dense stroma, we observed a higher rate of lung recurrence which may also be a plausible explanation for better outcomes in this cohort as our group previously reported that PDAC patients with lung recurrence had a more favorable clinical course compared to liver recurrence [30]. Consistently, another study reported that increased alpha-smooth muscle actin (αSMA) expression which leads to dense stroma may predict better survival outcomes in PDAC patients following surgical resection [23].

PCSCs have 100-fold increased tumorigenic potential as compared to cancer cells lacking stem cell characteristics [31]. CD44^+^ ESA^+^ CD24^+^ cancer cells demonstrate characteristics of stem cells including increased developmental signaling pathway (including SHH) leading to chemoresistance [32–34]. In our study, however, we did not identify any correlation with survival outcomes and PCSC markers including CD44 and ESA. That may in part be related to the small size of our cohort and selection of patients who underwent surgical resection. Notably, in contrast, to the report by Li et al., we also observed more histologically undifferentiated tumors with the expression of CD44 regardless of expression status of ESA (Table 1S), [32]. This finding supports the theory of epithelial-mesenchymal transition in which cancer cells gain more mesenchymal features and surface markers such as CD44 as they acquire stemness features with poor histologic differentiation. At this time, it is unclear if PCSCs may lead to increased distant metastasis with their mesenchymal features [35]. Conversely, in our study, we identified a significantly higher frequency of loose stroma in patients with enriched stem cell markers particularly with CD44 expression and patients with loose stroma had higher cumulative incidences of local recurrence 3-year post-surgery as compared to patients with dense stroma (Table 3). This finding suggests that CD44+ PCSC may have a direct effect on stroma differentiation and that they may impact local invasion over distant metastasis. Our study is limited with the small size of the patient cohort, the retrospective nature of the study, the selection of only surgically resected PDAC patients, the use of a single-institution database, and lack of detailed information regarding systemic therapy which may have influenced outcomes. Further studies with larger cohorts are warranted to better understand the mechanistic relationship between PCSC and tumor stroma and the effect on recurrence patterns in PDAC individuals who undergo surgical resection.

## Conclusion

Summing up, in our study we identified a relationship between PDAC stroma and PCSC markers with an increased incidence of loose stroma in PDAC expressing PCSC markers, particularly CD44. We also identified that PDAC stroma may have an impact on recurrence patterns and overall survival in PDAC patients who underwent surgical resection of their primary tumor. Interestingly, contrary to the hypothesis that highly desmoplastic tumor stroma may lead to adverse outcomes, patients with loose stroma had a worse clinical course suggesting that dense stroma may provide a physical barrier function to restrain the primary tumor and reduce local invasion. These findings collectively hypothesize that strategies targeting dense PDAC stroma may not improve outcome and speculatively may incur a negative outcome [18, 36].

## Supplementary Information

**Additional file 1: Figure 1S.** Staining intensity for CD44 and ESA was scored as 0, none (A); 1, weak (B); 2, moderate (C); 3, strong (D).

**Additional file 2: Table 1S.** Distribution of tumor grade by cancer stem cell markers.

## Data Availability

The datasets used and/or analyzed during the current study are available from the corresponding author on reasonable request.

## Abbreviations

PCSC: Pancreatic stem cell markers
PDAC: Pancreatic ductal adenocarcinoma
ESA: Epithelial-specific antigen
OS: Overall survival
RFS: Relapse-free survival
FOLFIRINOX: 5-Fluorouracil, leucovorin, oxaliplatin, irinotecan
MMR-D: Mismatch repair-deficient
PARP: Poly ADP ribose polymerase
SHH: Sonic hedgehog pathway
MSK: Memorial Sloan Kettering
αSMA: alpha-smooth muscle actin
