# Supplementary figures and images for: Pancreatic cancer stem cells may define tumor stroma characteristics and recurrence patterns in pancreatic ductal adenocarcinoma
Source: BMC Cancer. 2021 Apr 9;21:385. doi: 10.1186/s12885-021-08123-w (PMC8034174; doi:10.1186/s12885-021-08123-w)

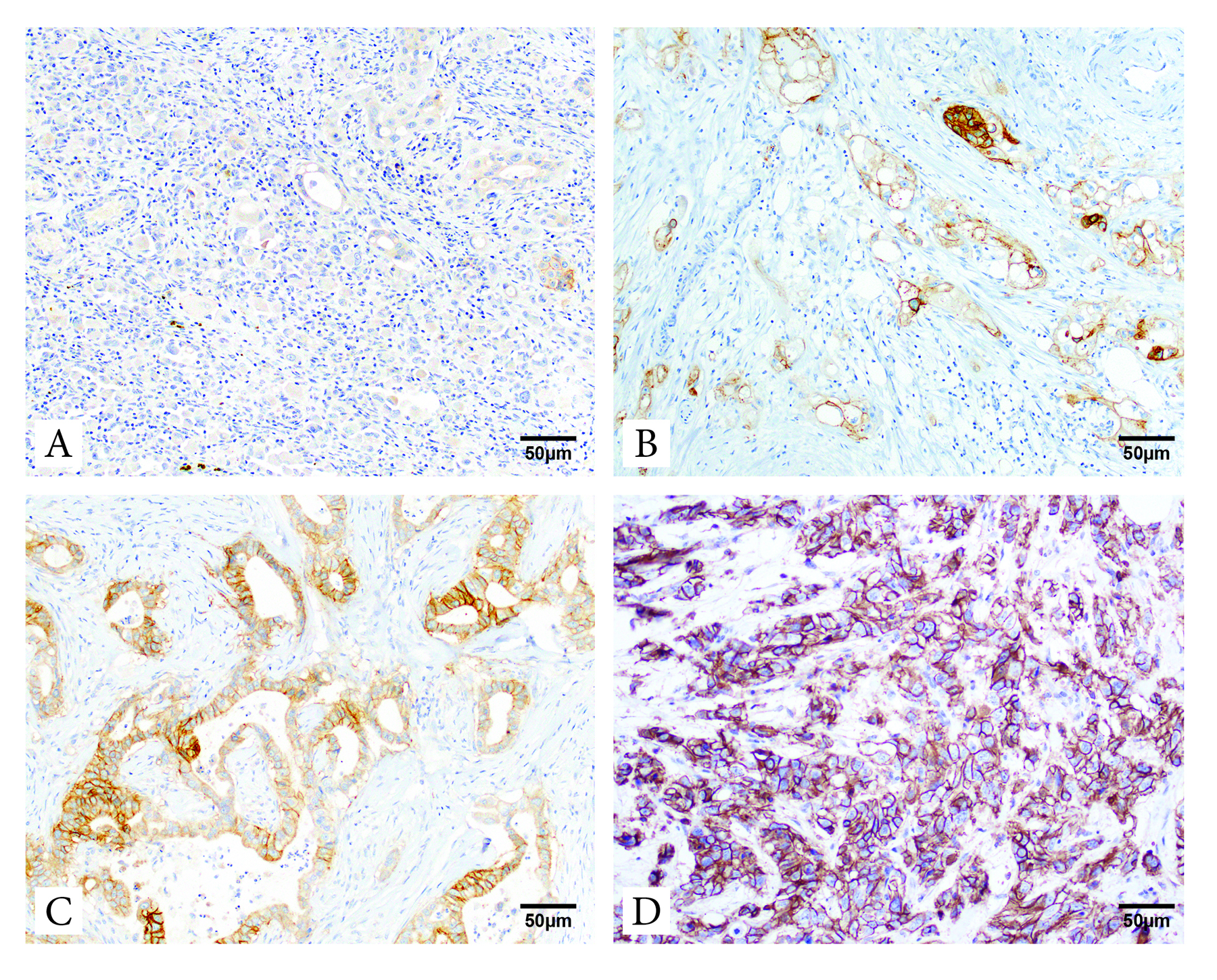

Supplement: Supplementary file 1 — Additional file 1: Figure 1S. Staining intensity for CD44 and ESA was scored as 0, none (A); 1, weak (B); 2, moderate (C); 3, strong (D). [file 12885_2021_8123_MOESM1_ESM.tif]
