# Supplementary material for: Pancreatic cancer stem cells may define tumor stroma characteristics and recurrence patterns in pancreatic ductal adenocarcinoma
Source: BMC Cancer. 2021 Apr 9;21:385. doi: 10.1186/s12885-021-08123-w (PMC8034174; doi:10.1186/s12885-021-08123-w)
Supplement: Supplementary file 2 — Additional file 2: Table 1S. Distribution of tumor grade by cancer stem cell markers. [file 12885_2021_8123_MOESM2_ESM.docx]

| **Table 1S. Distribution of tumor grade by cancer stem cell markers** | | | | |
| --- | --- | --- | --- | --- |
| **Histologic type** | **CD44+/ESA+ N (%)** | **CD44-/ESA+**  **N (%)** | **CD44+/ESA-**  **N (%)** | **CD44-/ESA-**  **N (%)** |
| **Well to moderately differentiated** | 3 (19%) | 27 (75%) | 5 (26%) | 5 (23%) |
| **Moderate to poorly differentiated** | 13 (81%) | 9 (25%) | 14 (74%) | 17 (77%) |
| **Total** | 16 | 36 | 19 | 22 |
